# Guide to the preparation of speech reports for implanted children: opinion of specialists

**DOI:** 10.1590/2317-1782/20232022177en

**Published:** 2023-12-22

**Authors:** Natália Barreto Frederigue-Lopes, Joice de Moura Silva, Flávia Custódio Pedroso de Souza, Marcela Beatriz Ricardo, Thais Corina Said de Angelo, Regina Tangerino de Souza Jacob, Adriane Lima Mortari Moret, Marina Morettin Zupelari

**Affiliations:** 1 Departamento de Fonoaudiologia, Faculdade de Odontologia de Bauru - FOB, Universidade de São Paulo - USP - Bauru (SP), Brasil.

**Keywords:** Cochlear Implantation, Audiologic Rehabilitation, Child, Report

## Abstract

**Purpose:**

To develop a guide for the preparation of speech-language reports of implanted children to be shared among speech-language pathologists of cochlear implant (CI) services and rehabilitation professionals.

**Methods:**

The Delphi method was used to select the relevant and fundamental items that should be included in the two versions proposed for the guide: Guide 1 - Speech-language reports provided by the CI services to rehabilitators, and Guide 2 - Speech-language reports provided by the rehabilitators to CI services. Twenty-one speech therapists specialized and with experience in cochlear implants and auditory rehabilitation participated in the discussion and judgment of the items during the selection rounds. Consensus was considered when the item reached agreement equal to or greater than 80% among participants, being selected to compose the two guides.

**Results:**

After the two rounds, 21 items from Guide 1 reached consensus among therapists, that is, more than 80% of them agreed that these items should be present in the report sent by the CI service. For Guide 2, 22 items analyzed by speech therapists working in CI services in the postoperative sector were selected in the second round.

**Conclusion:**

Based on the analysis of the two rounds, the “Guide for the preparation of speech-language pathology reports: intersection between CI service and rehabilitators” was developed. This material can be applied in the follow-up of implanted children, standardizing the information shared about the electronic device, evaluation results, monitoring of results and therapeutic process of this population.

## INTRODUCTION

Different health professionals are involved in the auditory rehabilitation process of hearing-impaired children who will receive or have received a cochlear implant (CI). The collaboration and partnership established between professionals who work with this population can optimize the results obtained by the child with the use of the CI and strengthen the family's engagement during the rehabilitation process^(1)^, leading to better health and functionality results for this population. A lack of interprofessional collaboration can drive to impaired speech and language development, academic progress, social interactions, vocational choices, and much more for that child^(2)^.

It is important that both the professionals who work in the CI service and the speech therapists responsible for the speech therapy of the child, before and after the CI surgery, have common goals and perspectives on their treatment, offering the child and their family continuous and collaborative care^(3)^. For this to happen, it is essential that these professionals have effective communication, allowing the exchange of information in an easy and understandable way for both specialties and thus favoring the identification of alert factors that may require some type of adjustment or modification in the intervention^(4)^.

A study by Davis et al.^(5)^ showed that, although audiologists and speech therapists who work with speech therapy consider the collaborative practice important, less than 70% have worked together in cases of hearing-impaired patients. For speech therapists working with therapy, the three main barriers for this partnership to occur were: access, time and communication, while the three main barriers found by audiologists were access, knowledge and attitude/perceptions. Regarding communication, this difficulty was related to the failure to establish efficient communication in terms of time and issues related to sharing information on patient outcomes and treatment. To improve this, the authors suggest the creation of a communication protocol to be shared via system or e-mail, for example.

Ward et al.^(6)^ investigated the communication between audiologists and speech therapists working with therapy regarding cochlear implant management and, although most therapists answered that they rarely communicate with audiologists, 62% consider this contact important. For the authors, both professionals could try to improve communication during the treatment and rehabilitation of hearing-impaired people. According to them, this bidirectional interaction can provide more current information about the features offered by the CI, allowing professionals to conduct a more effective treatment for this population.

In the United States, a group of surgeons, audiologists and computer scientists developed an online system to store and share audiological and demographic information about their patients pre and post CI surgery. The objective was to facilitate access to information on these patients, so that standardized data could be shared among members of the treatment network. This tool is in the implementation phase but, according to the authors, over time it will allow documenting patient data, thus enabling physicians, audiologists, speech therapists working with therapy and other professionals to ensure constant progress towards the goals of patients and their parents after implantation. In addition, this tool can streamline the coordination of care for this population, in addition to improving communication among professionals involved in their treatment and rehabilitation, regardless of the service they work with or their geographic location^(7)^.

In Brazil, due to its large territorial extension, it is common for rehabilitators to work in different and often distant places from the CI services. As a result, the exchange of information on the child tends to occur, in most cases, through the sharing of printed speech-language pathology reports. This communication tool between the rehabilitator and the CI service gathers relevant information and records about the technical aspects, the use of speech processor programs and the child's performance in the auditory speech perception tests. Furthermore, it provides parameters for comparing the results after implantation. However, tools for sharing standardized information between these services and professionals that can facilitate communication between them and guide collaborative work in their clinical practices with these children were not found in the literature.

In view of the above, the objective of this study was to develop a guide for the preparation of speech therapy reports of implanted children, to be shared with speech therapists at CI services and rehabilitators working with speech therapy for children using CI, to standardize the information on electronic device, evaluation results, monitoring of results, and therapeutic process of this population.

## METHOD

This prospective and cross-sectional study was approved by the Research Ethics Committee (REC) of the Bauru School of Dentistry - University of São Paulo (nº 1.712.713), with the Free and Informed Consent Form (FICF) waived by the REC and its replacement requested by invitation letter sent to participants via e-mail with proper guidelines regarding the objective and methodology of the research.

Two guides were proposed to develop the “Guide for the preparation of speech-language pathology reports: intersection between CI service and rehabilitators”:

1. Guide 1: Speech-Language pathology reports provided by the CI service to rehabilitators;
2. Guide 2: Speech-Language pathology reports provided by rehabilitators to CI services.

Speech therapists from all over Brazil were invited to participate in the study to allow different opinions regarding the main items that should be included in Guides 1 and 2 to be contemplated.

**Figure 1:**
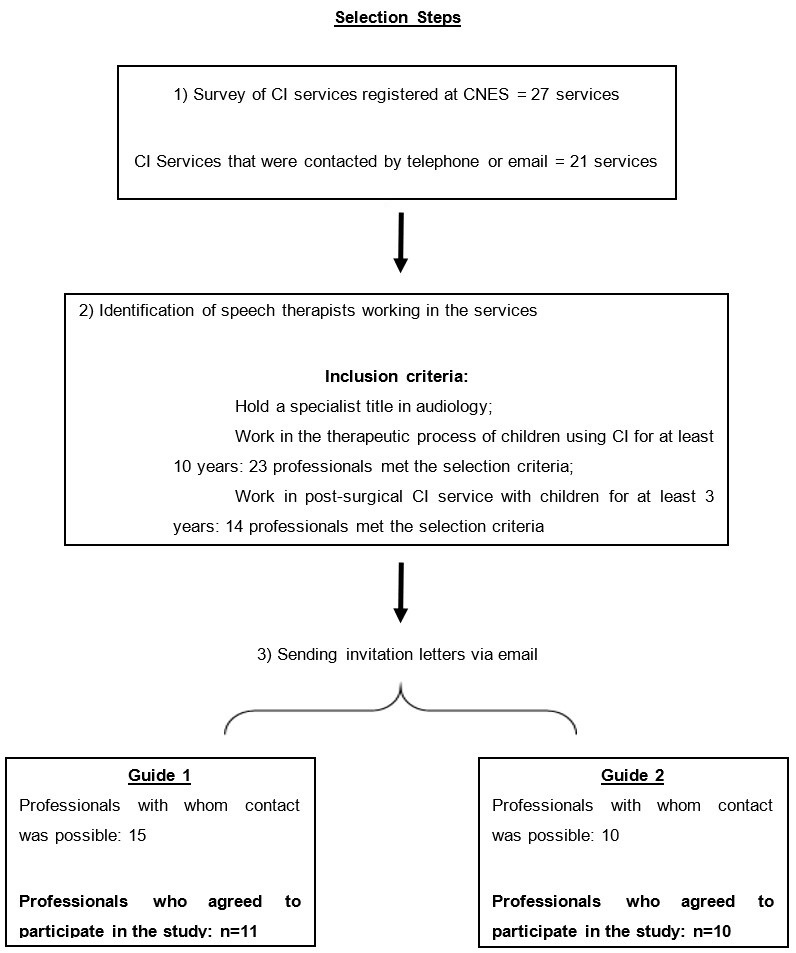
Steps for including expert participants to build Guides 1 and 2

Figure 1 summarizes the stages of inclusion of the speech therapists invited. After identifying the cochlear implant services registered in the Register of Health Establishments (CNES), telephone contact was made with these services to get the number of speech therapists working for at least three years in the post-surgery stage, and speech therapists working for at least 10 years with speech therapy with children using CI to later invite them to participate in the groups to develop the two guides. For both groups, the title of specialist in audiology was considered an inclusion criterion.

Contact was made with 21 CI services among the 27 surveyed. A total of 14 speech therapists working in the post-surgical stage and 23 speech therapists working with speech therapy for children using CI were contacted via email and invited to participate in the study.

A total of 21 speech therapists agreed to participate in the research:

- 11 speech therapists working with speech therapy for children using CI, who should select the items for Guide 1: Speech therapy reports provided by the CI service to rehabilitators and,
- 10 speech therapists working in the post-surgery stage, who should select the items for Guide 2: Speech therapy reports provided by rehabilitation professionals to CI services.

Table 1 shows the characterization of the participating speech therapists, by group, in terms of age, gender, time of experience and region of activity.

**Table 1:** Characterization of the experts participating in the construction of Guides 1 and 2

**Characterization**		**Speech therapists working in rehabilitation n=11**		**Speech therapists working in CI services n=10**	
		**n**	**%**	**n**	**%**
**Age**	30-40 years	3	27.28	2	20.00
	41-50 years	2	18.18	5	50.00
	51-60 years	4	36.36	2	20.00
	61-70 years	2	18.18	1	10.00
**Gender**	F	10	100.00	8	80.00
	M	0	0.00	2	20.00
**Time of work**	5-10 years	1	9.09	3	30.00
	11-15 years	3	27.27	4	40.00
	16-20 years	3	27.27	2	20.00
	21-30 years	4	36.36	1	10.00
**Region of work**	North	0	0.00	1	10.00
	Northeast	1	9.09	1	10.00
	Midwest	1	9.09	2	20.00
	Southeast	8	72.72	6	60.00
	South	1	9.09	0	0.00

Caption: CI = Cochlear implant; F = Female; M = Male

## DEVELOPMENT OF THE GUIDES

The Delphi technique was selected to identify the items that should be included in both guides^(8)^.

This technique allows a group of experts, residing in different locations, to interact anonymously through questionnaires to reach a consensus on a given topic^(9)^. The experts participate in a series of rounds by answering one or more specific questions via email, and after each round they receive feedback on the group's response to points of greatest and least agreement. The process of rounds is repeated to reduce the possibilities of answers/choices until the “consensus” is reached, that is, the divergences between them are discarded, leaving only the points of agreement. Generally, the consensus adopted in the studies varies from 55 to 100% of agreement in the answers, leaving this choice at the discretion of the researchers^(10)^.

For the initial elaboration of each guide, the researchers organized a list of items from existing and non-standard speech-language pathology reports and the main themes that should be included in the two guides, which included the information provided by CI services to rehabilitation professionals and vice versa.

The rounds took place through the Survey Monkey online platform. The participants received, via e-mail, a link to judge the relevance of the items based on the five-point Likert Scale: 1- strongly disagree; 2- disagree; 3- indifferent; 4- agree; 5- strongly agree. In this study, items chosen by 80% or more of the experts as 4 or 5 were recognized as consensus, and two rounds were used to select items from both guides^(11)^.

In addition to the speech therapists being asked to judge each item quantitatively (Likert scale), a space was added for suggestions and comments in each of them, which were analyzed after the rounds and considered for a new judgment in the following rounds.

- Round 1:

The electronic version of the previously prepared items was sent via e-mail to all the 21 specialist speech therapists who responded to the initial invitation.

Speech therapists judged the relevance of the items for Guide 1, and those who worked in the postoperative period for Guide 2, according to the five-point Likert scale.

After returning the responses, the researchers analyzed the frequency of responses given by the participants for each item and when the item obtained agreement above 80% in categories 4 and 5 of the Likert scale, consensus was recognized. In addition, speech therapists' comments were considered for the inclusion of new items in the second round.

- Round 2

The analysis of the list of items of the second round was conducted in the same way as the previous questionnaire. After organizing the items in both guides, the link was forwarded again via e-mail to the speech therapists so that they could judge the relevance of each item, offering the possibility of revising their opinion in this second round and allowing for new notes or confirmation of the answers provided previously.

The end of the second round followed the same procedures as the previous round, namely: survey of relevant data and consensus, and systematization of responses for the preparation of Guides 1 and 2.

The distribution time of the links via e-mail for the collection of responses in round 1, analysis of the results and collection of responses for round 2, was 30 and 60 days, respectively.

## RESULTS

Initially, Guide 1: Speech-Language Pathology reports provided by CI services to rehabilitation professionals, and Guide 2: Speech-language pathology reports provided by rehabilitation professionals to CI services, contained 16 and 25 items, respectively, distributed for the analysis of speech therapists in the first round.

After the first-round analysis, one item (1; 6%) in Guide 1 and four items (4; 16%) in Guide 2 were discarded, as less than 80% of judges in both groups considered these items necessary to compose the instruments.

In Guide 1, the item “receive the mapping report generated by the programming software” was deleted. In Guide 2, the items: type and degree of hearing loss, age at CI surgery and activation of electrodes, information on the existence of differences in the performance of auditory skills between the ears in the case of children using bilateral CI, and information on the use of telephone did not reach consensus among the speech therapists working in the postoperative period of the CI service, being excluded from the following round.

Thus, Guide 1 had 15 items selected by speech therapists in the first round, and Guide 2 had 21 items selected by speech therapists working in the postoperative period. Tables 2 and 3 summarize the items that reached consensus among speech therapists after the first round for Guides 1 and 2, respectively.

**Table 2:** Items that reached consensus during the first round of questionnaire application, Guide 1 (n=11)

**Questions**	**Totally disagree**		**Disagree**		**Neither agree nor disagree**		**Agree**		**Totally agree**	
	**n**	**%**	**n**	**%**	**n**	**%**	**n**	**%**	**n**	**%**
Identification	0	0	0	0	0	0	**4**	**36.36**	**7**	**63.64**
Follow-up frequency	0	0	0	0	0	0	**1**	**9.09**	**10**	**90.91**
Insertion of electrodes	0	0	0	0	1	9.09	**1**	**9.09**	**9**	**81.82**
Internal component and speech processor model	0	0	0	0	2	18.18	**2**	**18.18**	**7**	**63.64**
Operation and conditions of use of external components of CI	0	0	0	0	1	9.09	**3**	**27.27**	**7**	**63.64**
Internal component functionality	0	0	0	0	0	0	**4**	**36.36**	**7**	**63.64**
Speech coding strategy and frequency range for each map	0	0	0	0	1	9.09	**3**	**27.27**	**7**	**63.64**
Number of programs saved and Indication of use	0	0	0	0	0	0	**1**	**9.09**	**10**	**90.91**
Enabling volume and sensitivity control in the speech processor, how to use them	0	0	0	0	0	0	**0**	**0**	**11**	**100**
Sound and light alerts	0	0	0	0	1	9.09	**2**	**18.18**	**8**	**72.73**
Intercurrences during mapping	0	0	0	0	0	0	**2**	**18.18**	**9**	**81.82**
Use of each program by the patient (datalogging)	0	0	0	0	0	0	**1**	**9.09**	**10**	**90.91**
Free-field tonal audiometry with CI	0	0	0	0	0	0	**1**	**9.09**	**10**	**90.91**
Speech perception tests in silence and in noise	0	0	0	0	0	0	**2**	**18.18**	**9**	**81.82**
Indication of use of specific maps (remote microphone, etc.)	0	0	0	0	0	0	**2**	**18.18**	**9**	**81.82**

Caption: CI = Cochlear implant

**Table 3:** Items that reached consensus during the first round of questionnaire application, Guide 2 (n=10)

**Questions**	**Totally disagree**		**Disagree**		**Neither agree nor disagree**		**Agree**		**Totally agree**	
	**n**	**%**	**n**	**%**	**n**	**%**	**n**	**%**	**n**	**%**
Identification	0	0	0	0	0	0	**40**	**40**	**60**	**60**
Education	0	0	0	0	0	0	**50**	**50**	**50**	**50**
Etiology	0	0	10	10	10	10	**60**	**60**	**20**	**20**
Age at initiation of therapy	0	0	0	0	10	10	**0**	**0**	**90**	**90**
Age at initiation of service therapy	0	0	0	0	10	10	**40**	**40**	**50**	**50**
Use of HA prior to CI	0	0	0	0	10	10	**10**	**10**	**80**	**80**
Effective use of CI	0	0	0	0	0	0	**20**	**20**	**80**	**80**
Use of HA contralateral to CI	0	0	0	0	0	0	**30**	**30**	**70**	**70**
Auditory Skills Bilateral CI	0	0	10	10	0	0	**20**	**20**	**70**	**70**
Remote microphone	0	0	0	0	0	0	**40**	**40**	**60**	**60**
Data relative to MAPS - CI	0	0	10	10	0	0	**40**	**40**	**50**	**50**
Therapeutic Approach	0	0	0	0	0	0	**30**	**30**	**70**	**70**
Type of appointment	0	0	0	0	0	0	**50**	**50**	**50**	**50**
Number of appointments	0	0	0	0	10	10	**40**	**40**	**50**	**50**
Identification of the rehabilitating speech therapist	0	0	0	0	0	0	**50**	**50**	**50**	**50**
Family permeability	0	0	0	0	0	0	**30**	**30**	**70**	**70**
Child behavior	0	0	0	0	0	0	**30**	**30**	**70**	**70**
Language development	0	0	0	0	0	0	**20**	**20**	**80**	**80**
Language category	0	0	0	0	0	0	**20**	**20**	**80**	**80**
Auditory development	0	0	0	0	0	0	**20**	**20**	**80**	**80**
Auditory category	0	0	0	0	0	0	**20**	**20**	**80**	**80**

Caption: HA = Hearing aid; CI = Cochlear implant

Noteworthy, for Guide 1: Speech-language pathology reports provided by the CI service to rehabilitators, most of the items proposed in the first round reached a 100% consensus among the judging speech-language pathologists and therapists, that is, most of these participants agreed with the statement that the item should be included in the guide. The agreement of 80% to 90% among the speech therapists occurred for the items: insertion of electrodes, model of the internal component and speech processor, functioning and conditions of the external components of the CI, speech coding strategy and frequency range for each map and sound and light alerts (Table 2).

The same occurred for Guide 2: Speech-language pathology reports provided by rehabilitators to the CI services, for the items: etiology, age at the start of therapy, use of hearing aid before the CI, auditory skills before the bilateral CI, data on maps and service numbers (Table 3).

Participants did not express interest in modifying the items proposed in round 1 for the two guides.

The speech therapists' comments were systematized, resulting in the inclusion of seven new items in Guide 1 (etiology, age at diagnosis, age at first fitting of a hearing aid, number of electrodes, battery and battery compartment, how hearing thresholds were obtained and measurements used to get the map), and two (2) new items in Guide 2 (intercurrences with the device and relevant medical aspects).

Thus, in round 2, Guide 1 and Guide 2 had 22 and 23 items, respectively, and were sent back for the analysis of the speech therapists.

After receiving the questionnaires back, 21 items in Guide 1 reached consensus among speech therapists, that is, more than 80% of them agreed that these items should be present in the report sent by the CI service. For Guide 2, 22 items analyzed by speech therapists working in the postoperative period of CI services were selected in the second round.

In Guide 1, the items “Age at 1st fitting of Hearing AID (HA)” and “How the hearing thresholds were obtained” achieved the least consensus among therapists (18% of them did not agree that these items should be included in the guide). In Guide 2, all items were approved by more than 90% of speech therapists (Tables 4 and 5).

**Table 4:** Items that reached consensus during the second round of questionnaire application, Guide 1 (n=11)

**Questions**	**Totally disagree**		**Disagree**		**Neither agree nor disagree**		**Agree**		**Totally agree**	
	**n**	**%**	**n**	**%**	**n**	**%**	**n**	**%**	**n**	**%**
Etiology	0	0	0	0	1	9.09	**2**	**18.18**	**8**	**72.73**
Age at diagnosis	0	0	0	0	1	9.09	**3**	**27.27**	**7**	**63.64**
Age at first HA fitting	0	0	0	0	2	18.18	**4**	**36.36**	**5**	**45.46**
Follow-up frequency	0	0	0	0	1	9.09	**1**	**9.09**	**9**	**81.82**
Insertion of electrodes	0	0	0	0	0	0	**2**	**18.18**	**9**	**81.82**
Number of electrodes	0	0	0	0	0	0	**2**	**18.18**	**9**	**81.82**
Internal component and speech processor model	0	0	0	0	0	0	**2**	**18.18**	**9**	**81.82**
Operation and conditions of use of external components of CI	0	0	0	0	0	0	**3**	**27.27**	**8**	**72.73**
Battery and battery compartment	0	0	0	0	0	0	**3**	**27.27**	**8**	**72.73**
Functionality of internal component	0	0	0	0	0	0	**5**	**45.45**	**6**	**54.55**
Speech coding strategy and frequency range for each map	0	0	0	0	1	9.09	**4**	**36.36**	**6**	**54.55**
Number of programs saved and Indication of use	0	0	0	0	0	0	**1**	**9.09**	**10**	**90.91**
Enabling volume and sensitivity control in the speech processor, how to use them	0	0	0	0	0	0	**1**	**9.09**	**10**	**90.91**
Sound and light alerts	0	0	0	0	0	0	**1**	**9.09**	**10**	**90.91**
Intercurrences during mapping	0	0	0	0	0	0	**0**	**0**	**11**	**100**
Use of each program by the patient (datalogging)	0	0	0	0	0	0	**0**	**0**	**11**	**100**
Free-field tonal audiometry with CI	0	0	0	0	0	0	**3**	**27.27**	**8**	**72.73**
How the hearing thresholds were obtained	0	0	0	0	2	18.18	**4**	**36.36**	**5**	**45.46**
Speech perception tests in silence and in noise	0	0	0	0	0	0	**2**	**18.18**	**9**	**81.82**
Indication of use of specific maps (remote microphone, etc.)	0	0	0	0	0	0	**1**	**9.09**	**10**	**90.91**
Measures used to obtain the map	0	0	0	0	0	0	**4**	**36.36**	**7**	**63.64**

Caption: HA = Hearing aid; CI = Cochlear implant

**Table 5:** Items that reached consensus during the second round of questionnaire application, Guide 2 (n=10)

**Questions**	**Totally disagree**		**Disagree**		**Neither agree nor disagree**		**Agree**		**Totally agree**	
	**n**	**%**	**n**	**%**	**n**	**%**	**n**	**%**	**n**	**%**
Identification	10	10	0	0	0	0	**20**	**20**	**70**	**70**
Education	0	0	0	0	10	10	**20**	**20**	**70**	**70**
Age at initiation of therapy	0	0	0	0	10	10	**0**	**0**	**90**	**90**
Age at initiation of service therapy	0	0	0	0	10	10	**50**	**50**	**40**	**40**
Use of HA prior to CI	0	0	0	0	10	10	**40**	**40**	**50**	**50**
Effective use of CI	0	0	0	0	0	0	**20**	**20**	**80**	**80**
Use of HA contralateral to CI	0	0	0	0	0	0	**20**	**20**	**80**	**80**
Auditory skills bilateral CI	0	0	0	0	0	0	**20**	**20**	**80**	**80**
Remote microphone	0	0	0	0	0	0	**30**	**30**	**60**	**60**
Data relative to MAPS - CI	0	0	0	0	10	10	**40**	**40**	**50**	**50**
Therapeutic approach	0	0	0	0	0	0	**40**	**40**	**60**	**60**
Type of appointment	0	0	0	0	10	10	**40**	**40**	**50**	**50**
Number of appointments	0	0	0	0	0	0	**70**	**70**	**30**	**30**
Identification of the rehabilitating speech therapist	0	0	0	0	10	10	**20**	**20**	**70**	**70**
Family permeability	0	0	0	0	0	0	**40**	**40**	**60**	**60**
Child behavior	0	0	0	0	0	0	**40**	**40**	**60**	**60**
Language development	0	0	0	0	0	0	**20**	**20**	**80**	**80**
Language category	0	0	0	0	0	0	**30**	**30**	**70**	**70**
Auditory development	0	0	0	0	0	0	**10**	**10**	**90**	**90**
Auditory category	0	0	0	0	0	0	**20**	**20**	**80**	**80**
Intercurrences with the device	0	0	0	0	10	10	**40**	**40**	**50**	**50**
Relevant medical aspects	0	0	0	0	10	10	**60**	**60**	**30**	**30**

Caption: HA = Hearing aid; CI = Cochlear implant

Thus, following the analysis of the two rounds, the “Guide for the preparation of speech-language pathology reports: intersection between CI service and rehabilitators” was developed (Chart 1).

**Chart 1:** Guide for the preparation of speech-language pathology reports: intersection between CI service and rehabilitators

**Guide 1:** Guide Speech-language pathology reports provided by CI service to rehabilitators	**Guide 2:** Guide Speech-language pathology reports provided by rehabilitators to CI service
Child identification data:	Child identification data:
Name / Date of birth / Age / Etiology of hearing impairment / Age at audiological diagnosis / Age at 1st intervention with HA / Frequency of follow-up by the CI service	Name / Date of birth / Age / Education background / Age at start of auditory rehabilitation process / Age at start of rehabilitation process with the current service / current professional
Information on EXTERNAL COMPONENTS and CONDITIONS OF USE:	Information about ELECTRONIC DEVICES:
- Speech processor	- Use of HA prior to CI
- Cables	
- Transmitting antenna	
- Battery life	- Effectiveness of CI use
- Quantity of batteries	- Use of HA contralateral to CI (bimodal adaptation)
- Battery compartment	Information about Auditory Skills - in the case of bilateral CI, it is important to describe the ears separately.
- Assistant/remote control	Information about remote microphones
- Speech processor model and connectivity possibilities	Data referring to the responses observed with the different MAPs saved in the processor
Information about CI INTERNAL COMPONENT:	Therapeutic approach established
- Internal component model	Number of appointments/frequency
- Number of active electrodes	Identification of the speech therapist responsible for the rehabilitation
Information about electrodes insertion	Information about the permeability of the family to the therapeutic process
Datalogging Data	Information about the child's current general behavior
Electrode impedance telemetry results	Information about language development
Frequency range available for each map/program	Classification of hearing and language categories
Usual signal processing strategies	Information about device intercurrence
Information on the existence of specific maps for certain situations	Relevant medical aspects that can contribute to the follow-up at the CI service
Number of programs saved in the processor and usage indication	
Volume and sensitivity control: inform when enabled and how to use	
Information about alerts (sound/light)	
Information about complications during mapping	
Information on the results obtained in free field audiometry (when relevant)	

Caption: HA = Hearing aid; CI = Cochlear implant

## DISCUSSION

This study allowed the development of the “Guide for the preparation of speech-language pathology reports: intersection between CI service and rehabilitators” (Chart 1), a guideline material for the preparation of speech-language pathology reports applicable in the follow-up of implanted children to favor the monitoring of these children and to strengthen the communication channel between rehabilitator, family and CI service.

The guide included essential information both for speech therapists working in the auditory habilitation and rehabilitation process of children with cochlear implants (Guide 1) and for speech therapists working in CI services (Guide 2). Access to this information will enable an intersection between professionals and a more complete follow-up of children with CI, favoring the development of new care plans based on the individual needs of these children and their families. For parents of children using CI, this communication between professionals is extremely useful during their children's rehabilitation process^(12)^.

With the growing demand of children who receive CI devices in Brazil and around the world, the bond between the professionals involved throughout the rehabilitation process has changed, making it more difficult for the CI service and the therapist to establish/maintain frequent contact during discussions about the child for whom they share responsibility for treatment, unlike what happened during the first CI surgeries performed in the country. Close contact allowed the results obtained with the device to be further discussed, and both services and professionals to communicate more frequently.

Currently, it is a common practice in the care of children using CI and their families that the exchange of information between the service responsible for the CI and the therapist occurs both informally, through parents’ reports, and formally, through written reports. These two forms of information sharing do not allow relevant and standardized data to be monitored and/or followed up over time, which could cause discontinuity in the treatment process of the child using CI. In addition, reports with non-standard information may depend on the convenience of the professional or the family as to what information they wish to share.

The purpose of this instrument was to standardize information from speech-language reports shared between CI services and therapists of children using CI. In Brazil, no study was found with the proposal of a tool containing important information to be shared between CI services and therapists aimed at this specific population.

Internationally, the form “Information exchange: Supporting the Child with a Cochlear Implant”) was found on the website “Supporting Success for Children with Hearing Loss”^(13)^. The purpose of this form is to facilitate communication between professionals who work with children with cochlear implants and their families. The children’s guardians must ask each professional where the child receives the service to fill out the form, so they can evaluate and monitor their children’s performance, thus helping make any programming adjustments needed. It is recommended that professionals who work with children update this form monthly, bimonthly, and biannually.

The tool gathers information just like the guides proposed in this study, including child identification data, mapping data, CI use and progress in auditory and language skills, as well as contact information for professionals/services that are filling out the form. Although tool development was not explained, it can help to better understand how other groups act regarding the exchange of information among professionals.

In this study, the proposal of separate guides to be completed by therapists and speech therapists who work in the postoperative period of the CI service may allow more detailed data on the different practices to be collected. Although the identification data proposed for both guides are similar, allowing the understanding of the child's history regarding diagnosis, etiology, adaptation of different hearing devices/hearing aids and fundamental information related to therapeutic rehabilitation, such as age of initiation of auditory rehabilitation, the other topics do not have items in common in both guides.

For therapists, the item “receive the mapping report generated by the programming software” was not considered relevant, since the analysis of this report can be rather difficult for some professionals, although some reported that some information from the report should be included in Guide 1, which was done by the investigators.

This information is relevant, as studies have shown that, sometimes, the speech therapist working with children using CI needs preparation/training to work with the resources of cochlear implants, including troubleshooting, parts of the CI and general maintenance^(6)^. Since most of the items selected in Guide 1 require a basic/medium level of knowledge about how the CI works, it is essential that the services that share this guide with the children's therapists ensure that they master this knowledge so that the goals of use of the instrument are achieved.

On the other hand, items such as etiology, age at diagnosis, age at first fitting of a HA, number of electrodes, battery compartment, how hearing thresholds were obtained and measures used to get the map were included in Guide 1 based on the therapists' comments, allowing more specific data from the child's history and the functioning of the CI to be described in more detail for the therapist's knowledge.

In the study carried out by Athalye et al.^(14)^, it was suggested by the participants interviewed (parents, professionals and CI users) that CI services integrate more with other local services and improve communication and involvement with therapists, teachers and other professionals who participate in the network of care for this population. The participants would like to see more information exchange and more detailed reports on individual cases, in addition to joint work, as the guidelines received from the service or the therapist were often conflicting, making the scenario confusing and disturbing for families. Considering the lack of integration, the participants also reported that care for CI users seemed to be dictated by the needs of the service and not by the needs of the patient and their family.

The same considerations apply to information submitted by therapists to CI services. In Guide 2, the items: type and degree of hearing loss, age at CI surgery and activation of electrodes, information on the existence of differences in the performance of auditory skills between the ears in the case of a child using bilateral CI, and information on the use of the telephone did not reach consensus among the speech therapists who work in the postoperative period of the CI service, being excluded from the second round. Some participants reported that, because these items are already evaluated in such services, other priorities could be raised in the therapist's report. On the other hand, the speech therapists who work in the postoperative period considered it fundamental to include comments on the possible intercurrences with the device and the relevant medical aspects.

According to Ray et al.^(15)^, individual sessions with a speech therapist allow assessing the current skill level of each patient, which facilitates involvement through personalized discussions about goals, progress and expectations. In this way, the relevant detailed information included in the rehabilitation report (Guide 2) may provide a more specific view of the child's and family's routine, progress over time or the need to change specific aspects of the treatment.

The authors emphasize that auditory rehabilitation following cochlear implantation depends on skills and complementary knowledge of surgeons, speech therapists who work in CI services and therapists. Postoperative surgeons and speech therapists are responsible for managing the sensory information provided by the CI (CI implantation, activation and programming, in addition to tests of auditory skills and speech recognition performed in the services). However, according to the authors, such measures are static and do not provide detailed information on how the CI user is taking advantage of this resource in their daily lives. Thus, the therapist, in their routine with the patient, can assess the underlying cognitive-linguistic processing of the skills acquired after the CI surgery and provide information on the patient's performance, which can be used to guide expectations, goals and progress during the rehabilitation. Collectively, this information provides professionals, CI users and their families with a deeper understanding of individual needs and skills that contribute to self-reported speech recognition and performance outcomes^(15)^.

The information about the programs saved in the CI and the usage indication provided by the CI service to the therapist can help them establish the communication strategies in situations in which the CI user and their family report difficulties, guiding them on how to explore activities at home, in noisy situations and at school, for example. Once the therapist reviews how these guidelines are working, it is possible, through the report sent to the CI service, to provide comments on the topic “CI use”, regarding the child’s performance and, if needed, indication of program adjustments.

Thus, the “Guide for the preparation of speech-language pathology reports: intersection between CI service and rehabilitators” proves to be relevant for building a more effective communication between specialists in the area, in addition to favoring systematization in the process of monitoring children using CI assisted by different professionals in different regions of Brazil. Such communication is equally valuable for the formulation of guidance and counseling scripts, therapeutic plans and strategies adjusted to the needs of each child, with a view to achieving a more productive development of auditory skills, language and quality of life.

Putting the use of this tool into practice in the clinical routine of professionals working in the postoperative period and therapists of children using CI is the next step of this study, allowing the clinical validation of the instrument. In this way, it is expected to contribute to improving the quality of care aimed at the child population using CI, providing a more individualized and optimized treatment in terms of results.

## CONCLUSION

This study resulted in the development of the “Guide for the preparation of speech-language pathology reports: intersection between CI service and rehabilitators”, which includes:

- Guide 1: Speech-language pathology reports provided by CI service to rehabilitators, and
- Guide 2: Speech-language pathology reports provided by rehabilitators to CI service.

This material is intended to be applied in the routine monitoring of implanted children, being shared between speech therapists from CI services and rehabilitators working with speech therapy for children using CI, to standardize information on the electronic device, evaluation results, monitoring of results and therapeutic process of this population.
