# Supplementary material for: Guide to the preparation of speech reports for implanted children: opinion of specialists
Source: Codas. 2023 Dec 22;36(1):e20220177. doi: 10.1590/2317-1782/20232022177en (PMC10750860; doi:10.1590/2317-1782/20232022177en)
Supplement: Translation

# Translation: Guia para a elaboração de relatórios fonoaudiológicos de crianças implantadas: opinião de especialistas

**DOI:** 10.1590/2317-1782/20232022177pt

**Authors:** Natália Barreto Frederigue-Lopes, Joice de Moura Silva, Flávia Custódio Pedroso de Souza, Marcela Beatriz Ricardo, Thais Corina Said de Angelo, Regina Tangerino de Souza Jacob, Adriane Lima Mortari Moret, Marina Morettin Zupelari

**Affiliations:** 1 Departamento de Fonoaudiologia, Faculdade de Odontologia de Bauru - FOB, Universidade de São Paulo - USP - Bauru (SP), Brasil.

**Keywords:** Implante Coclear, Reabilitação da Deficiência Auditiva, Criança, Relatório

## Abstract

**Objetivo:**

Desenvolver guia para elaboração de relatórios fonoaudiológicos de crianças implantadas para serem compartilhados entre fonoaudiólogos dos serviços de implante coclear (IC) e reabilitadores.

**Método:**

O método Delphi foi utilizado para selecionar os itens relevantes e fundamentais que deveriam constar nas duas versões propostas para compor o guia: Guia 1 - Relatórios fonoaudiológicos fornecidos pelo serviço de IC aos reabilitadores, e Guia 2 - Relatórios fonoaudiológicos fornecidos pelos reabilitadores aos serviços de IC. Vinte e um fonoaudiólogos especialistas e com experiência na área de implante coclear e de reabilitação auditiva participaram da discussão e do julgamento dos itens durante as rodadas de seleção. Considerou-se consenso quando o item obteve a concordância igual ou superior a 80% entre os participantes, sendo selecionados para comporem os dois guias.

**Resultados:**

Após as duas rodadas, 21 itens do Guia 1 obtiveram consenso entre os terapeutas, ou seja, mais de 80% deles concordaram que estes itens deveriam estar presentes no relatório enviado pelo serviço de IC. Para o Guia 2, 22 itens analisados pelos fonoaudiólogos atuantes em serviços de IC setor pós-operatório, foram selecionados na segunda rodada.

**Conclusão:**

A partir da análise das duas rodadas, foi desenvolvido o “Guia para a elaboração de relatórios fonoaudiológicos: intersecção entre serviço de IC e reabilitadores”. Este material pode ser aplicado na rotina de acompanhamento de crianças implantadas, padronizando as informações compartilhadas sobre o dispositivo eletrônico, resultados de avaliações, monitoramento dos resultados e processo terapêutico dessa população.

## INTRODUÇÃO

Diferentes profissionais da saúde estão envolvidos no processo de reabilitação auditiva da criança com deficiência auditiva que irá receber ou recebeu o implante coclear (IC). A colaboração e a parceria estabelecidas entre os profissionais que atuam no cuidado dessa população podem otimizar os resultados obtidos pela criança com o uso do IC, fortalecer o engajamento da família durante o processo de reabilitação^(1)^, traduzindo-se em melhores resultados de saúde e funcionalidade para esta população. Já a falta de colaboração interprofissional pode resultar em comprometimento do desenvolvimento da fala e da linguagem, progresso acadêmico, interações sociais, escolhas vocacionais e muito mais para essa criança^(2)^.

É importante que tantos os profissionais que atuam no serviço de implante coclear, quando os fonoaudiólogos responsáveis pela terapia fonoaudiológica da criança, antes e depois da cirurgia de IC, tenham objetivos e perspectivas comuns sobre o seu tratamento, oferecendo a criança e a família um atendimento contínuo e colaborativo^(3)^. Para que isso ocorra, é fundamental que estes profissionais tenham uma forma de comunicação efetiva que permita a troca de informações de maneira fácil e compreensível para ambas as especialidades, com o objetivo de favorecer a identificação de fatores de alerta que venham a necessitar de algum tipo de ajuste ou modificação da intervenção^(4)^.

Estudo realizado por Davis et al.^(5)^, mostrou que apesar de audiologistas e fonoaudiólogos que atuam com terapia fonoaudiológica considerarem a prática colaborativa entre eles importante, menos de 70% tem atuado em conjunto nos casos de pessoas com deficiência auditiva. Para os fonoaudiólogos atuantes com terapia, as três principais barreiras elencadas para que esta parceria ocorra foram: acesso, tempo e comunicação, enquanto as três principais barreiras encontradas pelos audiologistas foram acesso, conhecimento e atitude/percepções. Em relação a comunicação, esta dificuldade foi relacionada ao não estabelecimento de um modo de comunicação eficiente em termos de tempo e problemas relacionados ao compartilhamento de informações sobre resultados de pacientes e tratamento. Como sugestão para melhorar este aspecto, os autores indicam a criação de um protocolo de comunicação que possa ser compartilhado via sistema ou e-mail, por exemplo.

Ward et al.^(6)^ investigaram em seu estudo a comunicação entre audiologistas e fonoaudiólogos que atuam com terapia em relação ao gerenciamento do implante coclear e, embora a maioria dos terapeutas tenham respondido que raramente se comunica com um audiologista, 62% consideram importante esse contato. Para os autores, ambos profissionais poderiam se esforçar mais para se comunicar durante o tratamento e reabilitação das pessoas com deficiência auditiva. Segundo eles, essa interação bidirecional é capaz de fornecer informações mais atuais sobre os recursos oferecidos pelo IC e permite aos profissionais conduzirem um tratamento mais eficaz para esta população.

Nos Estados Unidos, um grupo de cirurgiões, audiologistas e cientistas da computação, desenvolveram um sistema on-line para armazenar e compartilhar informações audiológicas e demográficas sobre seus pacientes pré e pós cirurgia de IC. O objetivo foi facilitar o acesso as informações desses pacientes, de modo que os dados padronizados pudessem ser compartilhados entre os membros da rede de tratamento. A ferramenta criada está em fase de implementação mas, segundo os autores, ao longo do tempo permitirá documentar os dados dos pacientes, possibilitando aos médicos, audiologistas, fonoaudiólogos que atuam com terapia e outros profissionais garantam um progresso constante em direção aos objetivos dos pacientes e dos pais após a implantação. Além disso, esta ferramenta poderá agilizar a coordenação dos cuidados com esta população, além de melhorar a comunicação entre os profissionais envolvidos no seu tratamento e reabilitação, independente do serviço que atuam ou de sua localização geográfica^(7)^.

No Brasil, devido a sua grande extensão territorial, é comum que os reabilitadores trabalhem em locais diferentes e, muitas vezes, distantes dos serviços de IC. Em virtude disso, as trocas de informações sobre a criança tendem a ocorrer, em grande parte dos casos, por meio do compartilhamento de relatórios fonoaudiológicos impressos. Este instrumento de comunicação entre o reabilitador e o serviço de IC reúne informações e registros relevantes acerca dos aspectos técnicos, do uso dos programas do processador de fala e do desempenho da criança nos testes de percepção auditiva da fala. Ademais, fornece parâmetros de comparação dos resultados com o uso do dispositivo. Contudo, não foi encontrada na literatura ferramentas de compartilhamento de informações padronizadas entre estes serviços e profissionais, que possam facilitar a comunicação entre eles e nortear o trabalho colaborativo nas suas práticas clínicas com estas crianças.

Diante do exposto, o objetivo deste estudo foi desenvolver um guia para a elaboração de relatórios fonoaudiológicos de crianças implantadas, a ser compartilhado entre os fonoaudiólogos dos serviços de IC e os reabilitadores que atuam com terapia fonoaudiológica de crianças usuárias de IC, a fim de padronizar as informações sobre o dispositivo eletrônico, os resultados de avaliações, o monitoramento dos resultados e o processo terapêutico dessa população.

## MÉTODO

O estudo de caráter prospectivo e transversal, foi aprovado pelo Comitê de Ética em Pesquisa em Seres Humanos (CEP) da Faculdade de Odontologia de Bauru da Universidade de São Paulo (parecer nº 1.712.713), com Termo de Consentimento Livre e Esclarecido (TCLE) dispensado pelo CEP, sendo solicitada a substituição por carta convite, enviada aos participantes via e-mail, com devidas as orientações relativas ao objetivo e a metodologia da pesquisa.

Para desenvolver o “Guia para a elaboração de relatórios fonoaudiológicos: intersecção entre serviço de IC e reabilitadores”, dois guias foram propostos:

1. Guia 1: Relatórios fonoaudiológicos fornecidos pelo serviço de IC aos reabilitadores;
2. Guia 2: Relatórios fonoaudiológicos fornecidos pelos reabilitadores aos serviços de IC.

Fonoaudiólogos de todo o Brasil foram convidados a participar do estudo, a fim de permitir que diferentes opiniões a respeito dos principais itens que deveriam ser incluídos nos Guias 1 e 2 fossem contempladas.

A Figura 1 resume as etapas de inclusão dos fonoaudiólogos convidados. Após a identificação dos serviços de implante coclear registrados no Cadastro de Estabelecimentos de Saúde (CNES), o contato telefônico foi realizado com estes serviços, a fim de levantar o número de fonoaudiólogos que trabalham há no mínimo três anos na etapa pós-cirúrgica, e fonoaudiólogos que atuam com terapia fonoaudiológica com crianças usuárias de IC há no mínimo 10 anos para, posteriormente, convidá-los para compor os grupos de construção dos dois guias. Para ambos grupos, o título de especialista em audiologia foi considerado como critério de inclusão.

**Figura 1:** Etapas de inclusão dos participantes especialistas para a construção do Guia nos Formatos 1 e 2

Dos 27 serviços de IC levantados, foi possível contato com 21. Destes, 14 fonoaudiólogos que trabalham na etapa pós-cirúrgica e 23 fonoaudiólogos que trabalham com terapia fonoaudiológica com crianças usuárias de IC foram contactados via e-mail e convidados a participar do estudo.

No total, 21 fonoaudiólogos concordaram em participar da pesquisa:

- 11 fonoaudiólogos que trabalham com terapia fonoaudiológica com crianças usuárias de IC, que deveriam selecionar os itens do Guia 1: Relatórios fonoaudiológicos fornecidos pelo serviço de IC aos reabilitadores e,
- 10 fonoaudiólogos que trabalham na etapa pós-cirúrgica, que selecionariam os itens que comporiam o Guia 2: Relatórios fonoaudiológicos fornecidos pelos reabilitadores aos serviços de IC.

A Tabela 1 apresenta a caracterização dos fonoaudiólogos participantes, por grupo, quanto a idade, gênero, tempo de atuação e região de atuação.

**Tabela 1:** Caracterização dos especialistas participantes da composição do Guia nos Formatos 1 e 2

**Caracterização**		**Fonoaudiólogos atuantes em reabilitação n=11**		**Fonoaudiólogos atuantes em serviços de IC n=10**	
		**n**	**%**	**n**	**%**
**Idade**	30 a 40 anos	3	27,28	2	20,00
	41 a 50 anos	2	18,18	5	50,00
	51 a 60 anos	4	36,36	2	20,00
	61 a 70 anos	2	18,18	1	10,00
**Sexo**	F	10	100,00	8	80,00
	M	0	0,00	2	20,00
**Tempo de atuação**	5 a 10 anos	1	9,09	3	30,00
	11 a 15 anos	3	27,27	4	40,00
	16 a 20 anos	3	27,27	2	20,00
	21 a 30 anos	4	36,36	1	10,00
**Região de atuação**	Norte	0	0,00	1	10,00
	Nordeste	1	9,09	1	10,00
	Centro Oeste	1	9,09	2	20,00
	Sudeste	8	72,72	6	60,00
	Sul	1	9,09	0	0,00

Legenda: IC = Implante Coclear; F = Feminino; M = Masculino

## DESENVOLVIMENTO DOS GUIAS

Para identificar os itens que deveriam constar nos dois guias, a técnica Delphi foi selecionada^(8)^.

Esta técnica permite que um grupo de especialistas, residentes em diferentes locais, interajam de forma anônima por meio de questionários, a fim de alcançar um consenso entre os mesmos sobre um determinado tema^(9)^. Os especialistas participam de uma série de rodadas respondendo a uma pergunta ou perguntas específicas por e-mail e, depois de cada rodada, recebem feedback sobre a resposta do grupo em relação aos pontos de maior e menor concordância. O processo de rodadas se repete com o objetivo de reduzir as possibilidades de respostas/escolhas até que o “consenso” seja alcançado, ou seja, as divergências entre eles são descartadas, restando apenas os pontos de concordância. Geralmente, o consenso adotado nos estudos varia de 55 a 100% de concordância nas respostas, sendo esta escolha reservada aos pesquisadores^(10)^.

Para a elaboração inicial de cada guia, os pesquisadores organizaram uma lista de itens, a partir do estudo de relatórios fonoaudiológicos já existentes e não padronizados, as principais temáticas que deveriam constar nos dois guias, que contemplassem as informações fornecidas pelos serviços de IC aos reabilitadores, e vice-versa.

As rodadas aconteceram pela plataforma online *Survey Monkey*. Os participantes receberam, via e-mail, um link para julgamento da relevância dos itens a partir da Escala de Likert de cinco pontos: 1- discordo totalmente; 2- discordo; 3- indiferente; 4- concordo; 5- concordo totalmente. Neste estudo foi reconhecido como consenso os itens que 80% ou mais dos especialistas escolheram como 4 ou 5 e definiu-se por realizar duas rodadas para seleção dos itens de ambos os guias^(11)^.

Além dos fonoaudiólogos serem solicitados a julgar cada item de forma quantitativa (escala de Likert), foi adicionado um espaço para sugestões e comentários em cada um deles, analisados após as rodadas e considerados para um novo julgamento nas rodadas seguintes.

- Rodada 1:

Foi enviada a versão eletrônica dos itens previamente elaborados por e-mail para cada um dos 21 fonoaudiólogos especialistas que responderam ao convite inicial.

Os fonoaudiólogos terapeutas julgaram a pertinência dos itens descritos no Guia 1 e os que trabalhavam no pós-operatório para o Guia 2, de acordo com a escala de Likert de cinco pontos.

Após a devolução das respostas, os pesquisadores analisaram a frequência das respostas fornecidas pelos participantes para cada item e quando este obteve concordância acima de 80% nas categorias 4 e 5 da escala de Likert, foi reconhecido consenso. Além disso, os comentários dos fonoaudiólogos foram considerados para a inclusão de novos itens na segunda rodada.

- Rodada 2

A análise da lista de itens da segunda rodada foi conduzida da mesma forma que o questionário anterior. Após a organização dos itens de ambos guias, foi novamente encaminhado aos fonoaudiólogos o *link* via *e-mail*, para que julgassem a relevância de cada item, dando a possibilidade de revisão de opinião nesta segunda rodada e, permitindo novos apontamentos ou confirmação das respostas anteriormente fornecidas.

O término da segunda rodada seguiu os mesmos procedimentos da rodada anterior, sendo: levantamento dos dados relevantes e consenso; e sistematização das respostas para a construção dos Guias 1 e 2.

O tempo de distribuição dos *links* via *e-mail* para a coleta das respostas na rodada 1, análise dos resultados e coleta de respostas para a rodada 2, foi de 30 e 60 dias, respectivamente.

## RESULTADOS

O Guia 1: Relatórios fonoaudiológicos fornecidos pelo serviço de IC aos reabilitadores, e o Guia 2: Relatórios fonoaudiológicos fornecidos pelos reabilitadores aos serviços de IC, foram compostos inicialmente por 16 e 25 itens, respectivamente distribuídos para análise dos fonoaudiólogos na primeira rodada.

Após a análise da primeira rodada, um (1; 6%) item do Guia 1 e quatro (4; 16%) itens do Guia 2 foram descartados, visto que menos de 80% dos juízes em ambos os grupos consideraram estes itens necessários para compor os instrumentos.

No Guia 1, o item “receber o relatório de mapeamento gerado pelo *software* de programação” foi excluído. No Guia 2, os itens: tipo e grau da perda auditiva, idade na cirurgia de IC e ativação dos eletrodos, informações sobre a existência de diferenças no desempenho das habilidades auditivas entre as orelhas no caso de criança usuária de IC bilateral, e informações sobre o uso do telefone, não obtiveram consenso entre os fonoaudiólogos que atuam no pós-operatório do serviço de IC, sendo excluídos da próxima rodada.

Desta forma, o Guia 1 teve 15 itens selecionados pelos fonoaudiólogos terapeutas na primeira rodada, e o Guia 2 teve 21 itens selecionados pelos fonoaudiólogos atuantes no pós-operatório. As Tabelas 2 e 3 sintetizam os itens que obtiveram consenso entre os fonoaudiólogos após a primeira rodada para os Guias 1 e 2, respectivamente.

**Tabela 2:** Itens que obtiveram consenso durante a primeira rodada de aplicação do questionário, Guia 1 (n=11)

**Questões**	**Discordo totalmente**		**Discordo**		**Indiferente**		**Concordo**		**Concordo totalmente**	
	**n**	**%**	**n**	**%**	**n**	**%**	**n**	**%**	**n**	**%**
Identificação	0	0	0	0	0	0	**4**	**36,36**	**7**	**63,64**
Frequência do acompanhamento	0	0	0	0	0	0	**1**	**9,09**	**10**	**90,91**
Inserção dos eletrodos	0	0	0	0	1	9,09	**1**	**9,09**	**9**	**81,82**
Modelo do componente interno e processador de fala	0	0	0	0	2	18,18	**2**	**18,18**	**7**	**63,64**
Funcionamento e condições dos componentes externos do IC	0	0	0	0	1	9,09	**3**	**27,27**	**7**	**63,64**
Funcionalidade do componente interno	0	0	0	0	0	0	**4**	**36,36**	**7**	**63,64**
Estratégia de codificação da fala e faixa de frequência para cada mapa	0	0	0	0	1	9,09	**3**	**27,27**	**7**	**63,64**
Número de programas salvos e indicação de uso	0	0	0	0	0	0	**1**	**9,09**	**10**	**90,91**
Habilitação do controle de volume e sensibilidade no processador de fala, como usá-los	0	0	0	0	0	0	**0**	**0**	**11**	**100**
Alertas sonoros e luminosos	0	0	0	0	1	9,09	**2**	**18,18**	**8**	**72,73**
Intercorrências durante o mapeamento	0	0	0	0	0	0	**2**	**18,18**	**9**	**81,82**
Uso de cada programa pelo paciente (*datalogging*)	0	0	0	0	0	0	**1**	**9,09**	**10**	**90,91**
Audiometria tonal em campo livre com IC	0	0	0	0	0	0	**1**	**9,09**	**10**	**90,91**
Testes de percepção de fala no silêncio e ruído	0	0	0	0	0	0	**2**	**18,18**	**9**	**81,82**
Indicação do uso de mapas específicos (Microfone remoto, etc.)	0	0	0	0	0	0	**2**	**18,18**	**9**	**81,82**

**Legenda:** IC = Implante coclear

**Tabela 3:** Itens que obtiveram consenso durante a primeira rodada de aplicação do questionário, Guia 2 (n=10)

**Questões**	**Discordo totalmente**		**Discordo**		**Indiferente**		**Concordo**		**Concordo totalmente**	
	**n**	**%**	**n**	**%**	**n**	**%**	**n**	**%**	**n**	**%**
Identificação	0	0	0	0	0	0	**40**	**40**	**60**	**60**
Escolaridade	0	0	0	0	0	0	**50**	**50**	**50**	**50**
Etiologia	0	0	10	10	10	10	**60**	**60**	**20**	**20**
Idade de início em terapia	0	0	0	0	10	10	**0**	**0**	**90**	**90**
Idade de início em terapia do serviço	0	0	0	0	10	10	**40**	**40**	**50**	**50**
Uso de DEAS prévio ao IC	0	0	0	0	10	10	**10**	**10**	**80**	**80**
Uso efetivo IC	0	0	0	0	0	0	**20**	**20**	**80**	**80**
Uso de DEAS contralateral ao IC	0	0	0	0	0	0	**30**	**30**	**70**	**70**
Habilidades Auditivas IC bilateral	0	0	10	10	0	0	**20**	**20**	**70**	**70**
Microfone remoto	0	0	0	0	0	0	**40**	**40**	**60**	**60**
Dados referentes aos MAPAS - IC	0	0	10	10	0	0	**40**	**40**	**50**	**50**
Abordagem Terapêutica	0	0	0	0	0	0	**30**	**30**	**70**	**70**
Tipo de atendimento	0	0	0	0	0	0	**50**	**50**	**50**	**50**
Número de atendimentos	0	0	0	0	10	10	**40**	**40**	**50**	**50**
Identificação do Fonoaudiólogo reabilitador	0	0	0	0	0	0	**50**	**50**	**50**	**50**
Permeabilidade Familiar	0	0	0	0	0	0	**30**	**30**	**70**	**70**
Comportamento da criança	0	0	0	0	0	0	**30**	**30**	**70**	**70**
Desenvolvimento da linguagem	0	0	0	0	0	0	**20**	**20**	**80**	**80**
Categoria de Linguagem	0	0	0	0	0	0	**20**	**20**	**80**	**80**
Desenvolvimento auditivo	0	0	0	0	0	0	**20**	**20**	**80**	**80**
Categoria de Audição	0	0	0	0	0	0	**20**	**20**	**80**	**80**

**Legenda:** DEAS = Dispositivo eletrônico de amplificação sonora; IC = Implante coclear

Nota-se que, para o Guia 1: Relatórios fonoaudiológicos fornecidos pelo serviço de IC aos reabilitadores, a maioria dos itens propostos na primeira rodada obtiveram consenso de 100% entre os fonoaudiólogos terapeutas julgadores, ou seja, a maioria destes participantes concordaram parcial ou com a afirmação de que o item deveria ser incluído no guia. A concordância de 80% a 90% entre os fonoaudiólogos terapeutas ocorreu para os itens: inserção dos eletrodos, modelo do componente interno e processador de fala, funcionamento e condições dos componentes externos do IC, estratégia de codificação da fala e faixa de frequência para cada mapa e alertas sonoros e luminosos (Tabela 2).

O mesmo ocorreu para o Guia 2: Relatórios fonoaudiológicos fornecidos pelos reabilitadores aos serviços de IC, para os itens: etiologia, idade de início da terapia, uso do dispositivo eletrônico de amplificação sonora antes do IC, habilidades auditivas antes do IC bilateral, dados referentes aos mapas e números de atendimentos (Tabela 3).

Não houve manifestação de interesse dos participantes sobre a modificação dos itens propostos na rodada 1 para os dois guias.

Os comentários dos fonoaudiólogos foram sistematizados, resultando na inclusão de sete novos itens no Guia 1 (etiologia, idade de diagnóstico, idade na primeira adaptação de dispositivo eletrônico de amplificação sonora, número de eletrodos, bateria e compartilhamento de bateria, como os limiares auditivos foram obtidos e medidas utilizadas para obtenção do mapa) e de dois (2) novos itens no Guia 2 (intercorrências com o dispositivo e aspectos médicos relevantes).

Desta forma, na rodada 2, o Guia 1 e o Guia 2 foram compostos por 22 e 23 itens, respectivamente, e reenviados para análise dos fonoaudiólogos.

Após o retorno dos questionários, 21 itens do Guia 1 obtiveram consenso entre os terapeutas, ou seja, mais de 80% deles concordaram que estes itens deveriam estar presentes no relatório enviado pelo serviço de IC. Para o Guia 2, 22 itens analisados pelos fonoaudiólogos atuantes no pós-operatório dos serviços de IC foram selecionados na segunda rodada.

Os itens do Guia 1 “Idade na 1ª adaptação do Dispositivo Eletrônico de Amplificação Sonora (DEAS)” e “Como os limiares auditivos foram obtidos” foram os que obtiveram menos consenso entre os terapeutas (18% deles não concordaram que estes itens deveriam ser incluídos no guia). No guia 2, todos os itens receberam aprovação de mais de 90% dos fonoaudiólogos (Tabelas 4 e 5).

**Tabela 4:** Itens que obtiveram consenso durante a segunda rodada de aplicação do questionário, Guia 1 (n=11)

**Questões**	**Discordo totalmente**		**Discordo**		**Indiferente**		**Concordo**		**Concordo totalmente**	
	**n**	**%**	**n**	**%**	**n**	**%**	**n**	**%**	**n**	**%**
Etiologia	0	0	0	0	1	9,09	**2**	**18,18**	**8**	**72,73**
Idade de diagnóstico	0	0	0	0	1	9,09	**3**	**27,27**	**7**	**63,64**
Idade na primeira adaptação de DEAS	0	0	0	0	2	18,18	**4**	**36,36**	**5**	**45,46**
Frequência do acompanhamento	0	0	0	0	1	9,09	**1**	**9,09**	**9**	**81,82**
Inserção dos eletrodos	0	0	0	0	0	0	**2**	**18,18**	**9**	**81,82**
Número de eletrodos	0	0	0	0	0	0	**2**	**18,18**	**9**	**81,82**
Modelo do componente interno e processador de fala	0	0	0	0	0	0	**2**	**18,18**	**9**	**81,82**
Funcionamento e condições de uso dos componentes externos do IC	0	0	0	0	0	0	**3**	**27,27**	**8**	**72,73**
Bateria e compartimento de bateria	0	0	0	0	0	0	**3**	**27,27**	**8**	**72,73**
Funcionalidade do componente interno	0	0	0	0	0	0	**5**	**45,45**	**6**	**54,55**
Estratégia de codificação da fala e faixa de frequência para cada mapa	0	0	0	0	1	9,09	**4**	**36,36**	**6**	**54,55**
Número de programas salvos e indicação de uso	0	0	0	0	0	0	**1**	**9,09**	**10**	**90,91**
Habilitação do controle de volume e sensibilidade no processador de fala, como usá-los	0	0	0	0	0	0	**1**	**9,09**	**10**	**90,91**
Alertas sonoros e luminosos	0	0	0	0	0	0	**1**	**9,09**	**10**	**90,91**
Intercorrências durante o mapeamento	0	0	0	0	0	0	**0**	**0**	**11**	**100**
Uso de cada programa pelo paciente (*datalogging*)	0	0	0	0	0	0	**0**	**0**	**11**	**100**
Audiometria tonal em campo livre com IC	0	0	0	0	0	0	**3**	**27,27**	**8**	**72,73**
Como os limiares auditivos foram obtidos	0	0	0	0	2	18,18	**4**	**36,36**	**5**	**45,46**
Testes de percepção de fala no silêncio e ruído	0	0	0	0	0	0	**2**	**18,18**	**9**	**81,82**
Indicação do uso de mapas específicos (Microfone remoto, etc.)	0	0	0	0	0	0	**1**	**9,09**	**10**	**90,91**
Medidas utilizadas para obtenção do mapa	0	0	0	0	0	0	**4**	**36,36**	**7**	**63,64**

**Legenda:** DEAS = Dispositivo eletrônico de amplificação sonora; IC = Implante coclear

**Tabela 5:** Itens que obtiveram consenso durante a segunda rodada de aplicação do questionário, Guia 2 (n=10)

**Questões**	**Discordo totalmente**		**Discordo**		**Indiferente**		**Concordo**		**Concordo totalmente**	
	**n**	**%**	**n**	**%**	**n**	**%**	**n**	**%**	**n**	**%**
Identificação	10	10	0	0	0	0	**20**	**20**	**70**	**70**
Escolaridade	0	0	0	0	10	10	**20**	**20**	**70**	**70**
Idade de início em terapia	0	0	0	0	10	10	**0**	**0**	**90**	**90**
Idade de início em terapia do serviço	0	0	0	0	10	10	**50**	**50**	**40**	**40**
Uso de DEAS prévio ao IC	0	0	0	0	10	10	**40**	**40**	**50**	**50**
Uso efetivo IC	0	0	0	0	0	0	**20**	**20**	**80**	**80**
Uso de DEAS contralateral ao IC	0	0	0	0	0	0	**20**	**20**	**80**	**80**
Habilidades Auditivas IC bilateral	0	0	0	0	0	0	**20**	**20**	**80**	**80**
Microfone remoto	0	0	0	0	0	0	**30**	**30**	**60**	**60**
Dados referentes aos MAPAS - IC	0	0	0	0	10	10	**40**	**40**	**50**	**50**
Abordagem Terapêutica	0	0	0	0	0	0	**40**	**40**	**60**	**60**
Tipo de atendimento	0	0	0	0	10	10	**40**	**40**	**50**	**50**
Número de atendimentos	0	0	0	0	0	0	**70**	**70**	**30**	**30**
Identificação do Fono reabilitador	0	0	0	0	10	10	**20**	**20**	**70**	**70**
Permeabilidade Familiar	0	0	0	0	0	0	**40**	**40**	**60**	**60**
Comportamento da criança	0	0	0	0	0	0	**40**	**40**	**60**	**60**
Desenvolvimento da linguagem	0	0	0	0	0	0	**20**	**20**	**80**	**80**
Categoria de Linguagem	0	0	0	0	0	0	**30**	**30**	**70**	**70**
Desenvolvimento auditivo	0	0	0	0	0	0	**10**	**10**	**90**	**90**
Categoria de Audição	0	0	0	0	0	0	**20**	**20**	**80**	**80**
Intercorrências com o dispositivo	0	0	0	0	10	10	**40**	**40**	**50**	**50**
Aspectos médicos relevantes	0	0	0	0	10	10	**60**	**60**	**30**	**30**

**Legenda:** DEAS = Dispositivo eletrônico de amplificação sonora; IC = Implante coclear

Assim, a partir da análise das duas rodadas, foi desenvolvido o “Guia para a elaboração de relatórios fonoaudiológicos: intersecção entre serviço de IC e reabilitadores” (Quadro 1).

**Quadro 1:** Guia para a elaboração de relatórios fonoaudiológicos: intersecção entre serviço de IC e reabilitadores

**Guia 1:** Guia Relatórios fonoaudiológicos fornecidos pelo serviço de IC aos reabilitadores	**Guia 2:** Guia Relatórios fonoaudiológicos fornecidos pelos reabilitadores aos serviços de IC
Dados de identificação da criança:	Dados de identificação da criança:
Nome/Data de nascimento/Idade/Etiologia da DA/Idade na época do diagnóstico audiológico/Idade na 1ª intervenção com DEAS/Frequência do acompanhamento pelo serviço de IC	Nome/Data de nascimento/Idade/Escolaridade/Idade de início no processo de reabilitação auditiva/Idade de início no processo de reabilitação no serviço atual/profissional atual
Informações sobre os COMPONENTES EXTERNOS e CONDIÇÕES DE USO:	Informações sobre os DISPOSITIVOS ELETRÔNICOS:
- Processador de fala	- Uso de DEAS prévio ao IC
- Cabos	- Efetividade de uso do IC
- Antena transmissora	- Uso de DEAS contralateral ao IC (adaptação bimodal)
- Duração da bateria	Informações sobre as Habilidades Auditivas - no caso de IC bilateral, importante descrever os ouvidos separadamente.
- Quantidade de baterias	
- Compartimento de bateria	Informações sobre microfones remotos
- Assistente/controle remoto	
- Modelo do processador de fala e possibilidades de conectividade	Dados referentes às respostas observadas com os diferentes MAPAS salvos no processador
Informações sobre o COMPONENTE INTERNO DO IC:	Abordagem Terapêutica estabelecida
- Modelo do componente interno	Número de atendimentos/frequência
- Número de eletrodos ativos	Identificação do Fonoaudiólogo responsável pela reabilitação
Informação sobre a inserção dos eletrodos	Informações sobre a permeabilidade da família ao processo terapêutico
Dados do *Datalogging*	Informações sobre o comportamento geral atual da criança
Resultados de telemetria de impedância dos eletrodos	Informações sobre o desenvolvimento da linguagem
Faixa de frequência disponível para cada mapa/programa	Classificação das categorias de audição e linguagem
Estratégias de processamento do sinal usuais	Informações sobre intercorrências com o dispositivo
Informar se há mapas específicos para determinadas situações	Aspectos médicos relevantes que possam contribuir para o atendimento do acompanhamento no serviço de IC
Número de programas salvos no processador e indicação de uso	
Controle de volume e sensibilidade: informar quando habilitados e como usar	
Informação sobre alertas (sonoros/luminosos)	
Informações sobre intercorrências durante o mapeamento	
Informações sobre os resultados obtidos na audiometria em campo livre (quando pertinentes)	
Informações sobre os resultados de percepção da fala nas condições silêncio e ruído (quando pertinentes)	

**Legenda:** DA = Deficiência Auditiva; DEAS = Dispositivo eletrônico de amplificação sonora; IC = Implante coclear

## DISCUSSÃO

A realização deste estudo permitiu o desenvolvimento do “Guia para a elaboração de relatórios fonoaudiológicos: intersecção entre serviço de IC e reabilitadores” (Quadro 1), um material de orientação para a elaboração de relatórios fonoaudiológicos aplicável na rotina de acompanhamento de crianças implantadas, com o intuito de favorecer o monitoramento destas crianças e fortalecer o canal de comunicação entre reabilitador, família e serviço de IC.

O guia incluiu informações fundamentais, tanto para os fonoaudiólogos que trabalham no processo de habilitação e de reabilitação auditivas de crianças com implante coclear (Guia 1), quanto para os fonoaudiólogos atuantes em serviços de IC (Guia 2). O acesso a estas informações possibilitará uma intersecção entre os profissionais e um acompanhamento mais completo de crianças com IC, favorecendo a elaboração de novos planejamentos de atendimentos baseados nas necessidades individuais destas crianças e suas famílias. Para os pais das crianças usuárias de IC, esta comunicação entre os profissionais é extremamente útil durante o processo de reabilitação de seus filhos^(12)^.

Com a crescente demanda de crianças que recebem dispositivos de IC no Brasil e no mundo, o vínculo entre os profissionais envolvidos durante todo o processo de reabilitação tem se modificado, sendo mais difícil para o serviço de IC e o terapeuta estabelecer/manter contato frequente entre eles durante as discussões sobre a criança a qual compartilham a responsabilidade do tratamento, diferentemente do que acontecia durante as primeiras cirurgias de IC realizadas no país. Este contato próximo permitia que os resultados obtidos com o dispositivo fossem mais debatidos e que ambos, serviços e profissionais, se comunicassem mais frequentemente.

Atualmente, é comum na prática de atendimento com as crianças usuárias de IC e suas famílias que as trocas de informações entre o serviço responsável pelo implante coclear e o terapeuta ocorram tanto de maneira informal, por meio dos relatos dos pais, ou formal, por meio de relatórios escritos. As duas formas de compartilhamento de informações não permitem que os dados relevantes e padronizados sejam monitorados e/ou acompanhados ao longo do tempo, podendo provocar a descontinuidade no processo de tratamento da criança usuária de IC. Além disso, relatórios com informações não padronizadas por muitas vezes podem depender da conveniência do profissional ou da família sobre qual(is) informação(es) desejam compartilhar.

A proposta do desenvolvimento deste instrumento foi exatamente padronizar as informações dos relatórios fonoaudiológicos compartilhados entre os serviços de IC e terapeutas das crianças usuárias de IC. Nenhum estudo no Brasil foi encontrado com a proposta de uma ferramenta contendo quais são as informações importantes de serem compartilhadas entre os serviços de IC e terapeutas voltada a esta população específica.

Internacionalmente, foi encontrado o formulário “Trocas de informações: Apoiando a criança com implante coclear (tradução literal para *“Information exchange: Supporting the Child with a Cochlear Implant”*) no site “*Supporting Sucess for Children with Hearing Loss*”^(13)^. Este formulário tem como objetivo facilitar a comunicação entre profissionais que atendem a criança com implante(s) coclear(res) e seus familiares. Os responsáveis pela criança devem solicitar a cada profissional onde a criança recebe o serviço que preencham o formulário, para que possam avaliar e monitorar o seu desempenho, auxiliando nos ajustes de programação. Eles recomendam que os profissionais que atuam com as crianças realizem atualizações mensais, bimestrais e semestrais deste formulário.

Esta ferramenta reúne informações como os guias propostos neste estudo, como dados de identificação da criança, dados de mapeamento, uso do IC e seu progresso quanto as habilidades auditivas e de linguagem, além de informações de contato sobre os profissionais/serviços que estão preenchendo. Apesar de não ter sido relatado como foi desenvolvido, pode auxiliar a compreender melhor como outros grupos atuam em relação a trocas de informações entre profissionais.

Neste estudo, a proposta de guias separados para serem preenchidos pelos terapeutas e por fonoaudiólogos que atuam no pós-operatório do serviço de IC pode permitir que dados mais detalhados sobre as diferentes práticas sejam coletados. Apesar dos dados de identificação propostos para ambos os guias serem semelhantes, permitindo a compreensão do histórico da criança quanto ao diagnóstico, etiologia, adaptação dos diferentes dispositivos auditivos/auxiliares da audição e informações fundamentais relativas à reabilitação terapêutica, como idade de início de reabilitação auditiva, os demais tópicos não possuem itens em comum nos dois guias.

Para os terapeutas, o item como “receber o relatório de mapeamento gerado pelo *software* de programação” não foi considerado importante, visto que a análise deste relatório pode ser mais difícil para alguns profissionais, apesar de alguns relatarem que algumas informações dele deveriam ser incluídas no Guia 1, o que foi realizado pelos investigadores.

Esta informação é relevante, pois estudos têm demonstrado que por vezes, o fonoaudiólogo que trabalha em terapia com crianças usuárias de IC precisa de uma preparação/treinamento para atuar com os recursos dos implantes cocleares, incluindo tópicos como: solução de problemas, partes do IC e manutenção geral^(6)^. Visto que a maioria dos itens selecionados no Guia 1 requer um nível básico/médio de conhecimento sobre o funcionamento do IC, é fundamental que os serviços que compartilharem este guia com os terapeutas das crianças, garantam que eles se apropriem destes conhecimentos, para que os objetivos de uso do instrumento sejam alcançados.

Por outro lado, itens como etiologia, idade de diagnóstico, idade na primeira adaptação de DEAS, número de eletrodos, e compartilhamento de bateria, como os limiares auditivos foram obtidos e medidas utilizadas para obtenção do mapa foram incluídos no Guia 1, a partir dos comentários dos terapeutas, permitindo que dados mais específicos da história da criança e de funcionamento do IC pudessem ser descritos com mais detalhes para conhecimento do terapeuta.

No estudo realizado por Athalye et al.^(14)^, foi sugerido pelos participantes entrevistados (pais, profissionais e usuários de IC) que os serviços de IC se integrassem mais com outros serviços locais e melhorassem a comunicação e o envolvimento com os terapeutas, professores e outros profissionais que participam da rede de atendimento dessa população. Os participantes gostariam que ocorresse mais trocas de informações e relatórios melhor detalhados sobre os casos individuais, além de um trabalho conjunto, pois muitas vezes as orientações recebidas do serviço ou do terapeuta eram conflitantes, tornando o cenário confuso e perturbador para as famílias. Frente a falta de integração, os participantes relataram ainda que o atendimento aos usuários de IC parecia ditado pelas necessidades do serviço e não pelas necessidades do paciente e família.

O mesmo podemos considerar em relação às informações enviadas pelos terapeutas ao serviço de IC. No Guia 2, os itens: tipo e grau da perda auditiva, idade na cirurgia de IC e ativação dos eletrodos, informações sobre a existência de diferenças no desempenho das habilidades auditivas entre as orelhas no caso de criança usuária de IC bilateral e as informações sobre o uso do telefone, não obtiveram consenso entre os fonoaudiólogos que atuam no pós-operatório do serviço de IC, sendo excluídos da segunda rodada. Alguns participantes comentaram que, pelo fato destes itens já serem avaliados nestes serviços, outras prioridades poderiam ser levantadas no relatório do terapeuta. Por outro lado, os fonoaudiólogos que atuam no pós-operatório consideraram fundamental incluir comentários sobre as possíveis intercorrências com o dispositivo e os aspectos médicos relevantes.

Segundo Ray et al.^(15)^, as sessões individuais com um fonoaudiólogo oferecem a oportunidade de avaliação do nível de habilidade atual de cada indivíduo, o que facilita o envolvimento por meio de discussões personalizadas sobre metas, progresso e expectativas. Desta forma, as importantes informações detalhadas incluídas no relatório do reabilitador (Guia 2) poderá fornecer uma visão mais específica da rotina desta criança e família, avanços ao longo do tempo ou necessidade de alterar algum aspecto específico do tratamento.

Estes autores reforçam que a reabilitação auditiva após o IC depende de habilidades e de conhecimentos complementares de cirurgiões, fonoaudiólogos que atuam nos serviços de IC e terapeutas. Cirurgiões e fonoaudiólogos do pós-operatório são responsáveis por gerir a informação sensorial proporcionada pelo IC (implantação do IC, ativação e programação, além de testes de habilidades auditivas e reconhecimentos de fala realizados nos serviços). Entretanto, segundo os autores, tais medidas são estáticas e não fornecem informações detalhadas como o usuário de IC está aproveitando este recurso no seu dia a dia. Desta forma, o terapeuta em sua rotina com o paciente pode avaliar o processamento cognitivo-linguístico subjacentes das habilidades adquiridas após a cirurgia de IC, podendo fornecer informações sobre o desempenho do paciente, que podem ser usadas para orientar expectativas, metas e progressos durante a reabilitação. Coletivamente, estas informações oferecem aos profissionais, usuários de IC e família, uma maior compreensão das necessidades e das habilidades individuais que contribuem para os resultados de reconhecimento de fala e desempenho autorrelatados^(15)^.

A informação sobre os programas salvos no IC e indicação de uso fornecida pelo serviço de IC ao terapeuta pode auxiliá-lo a determinar o uso de estratégias de comunicação em situações que o usuário de IC e família relatam dificuldades, orientando-os sobre como explorar melhor as atividades em casa, em situações de ruído e escola, por exemplo. Uma vez que o terapeuta revisa como estas orientações estão funcionando, é possível por meio do relatório enviado ao serviço de IC, o fornecimento de comentários no tópico “uso do IC”, a respeito do desempenho da criança e, se necessário, a indicação do ajuste de programas.

Assim, a construção do “Guia para a elaboração de relatórios fonoaudiológicos: intersecção entre serviço de IC e reabilitadores” mostra-se relevante para a construção de uma comunicação mais efetiva entre os especialistas da área, além disso favorece a sistematização no processo de acompanhamento de crianças usuárias de IC atendidas por diferentes profissionais em variadas regiões do Brasil. Tal comunicação é igualmente valiosa para a formulação de roteiros de orientação e de aconselhamento, planejamentos terapêuticos e estratégias ajustadas às necessidades de cada criança, com vistas ao alcance de um desenvolvimento mais produtivo das habilidades auditivas, de linguagem e de qualidade de vida.

Colocar em prática o uso desta ferramenta na rotina clínica dos profissionais atuantes no pós-operatório e terapeutas de crianças usuárias de IC é o próximo passo deste estudo, permitindo a validação clínica do instrumento. Espera-se desta forma contribuir para melhorar a qualidade do atendimento voltado à população infantil usuária de IC, proporcionando um tratamento mais individualizado e otimizado quanto aos resultados.

## CONCLUSÃO

Este estudo resultou no desenvolvimento do material “Guia para a elaboração de relatórios fonoaudiológicos: intersecção entre serviço de IC e reabilitadores”, composto por:

- Guia 1: Relatórios fonoaudiológicos fornecidos pelo serviço de IC aos reabilitadores, e
- Guia 2: Relatórios fonoaudiológicos fornecidos pelos reabilitadores aos serviços de IC.

Almeja-se que este material seja aplicado na rotina de acompanhamento de crianças implantadas, sendo compartilhados entre fonoaudiólogos dos serviços de IC e reabilitadores que atuam com terapia fonoaudiológica de crianças usuárias de IC, a fim de padronizar as informações sobre o dispositivo eletrônico, resultados de avaliações, monitoramento dos resultados e processo terapêutico dessa população.
